# Self-cleaning of Surfaces: the Role of Surface Wettability and Dust Types

**DOI:** 10.1038/srep38239

**Published:** 2016-12-05

**Authors:** Yun-Yun Quan, Li-Zhi Zhang, Rong-Hui Qi, Rong-Rong Cai

**Affiliations:** 1 Key Laboratory of Enhanced Heat Transfer and Energy Conservation of Education Ministry, School of Chemistry and Chemical Engineering, South China University of Technology, Guangzhou 510640, China; 2 State Key Laboratory of Subtropical Building Science, South China University of Technology, Guangzhou 510640, China

## Abstract

The self-cleaning property is usually connected to superhydrophobic surfaces (SHSs) where the dust particles can be easily removed by the rolling motion of droplets. It seems that superhydrophobicity (its durability is questionable nowadays) is a necessity. However here, it is disclosed that self-cleaning can also be realized on an ordinary surface by droplet impinging. The effects of surface wettability and the types of dust particles are considered. The self-cleaning is realized by two steps: (1) the pickup of particles by the water-air interface of an impinging droplet, (2) the release of the impinging droplets from the surface. It can be observed that only the trailing edges of the droplets can pick up particles when the droplets recoil from the inclined surfaces. The hydrophilic surface can also achieve self-cleaning under some conditions. This interesting finding may be helpful for the successful implementation of self-cleaning with common surfaces.

Dust pollution poses a widespread adverse effect on life and production, varying from buildings to solar panels^1^. Common cleaning methods have many problems, such as the high maintenance cost and the use of chemical detergents^2^. So the development of environmental-friendly self-cleaning surfaces which could keep themselves dust-proof in nature (like with the help of rainfall washing), has great practical significance.

Superhydrophobic surfaces (SHSs), having a water contact angle (CA) greater than 150° and a sliding angle (SA) smaller than 10°, are able to achieve self-cleaning by the so-called “lotus effect”^3^. There are two conditions for self-cleaning: (1) the pickup of dust particles by droplets, (2) the rolling off of droplets from surfaces at small inclinations. The pickup of dust particles is due to the attachment of the particles to the water-air interface^4^,^5^. Several forces should be considered including the interface force between the water-air interface and the particle^6^, the adhesion force between the solid surface and the particles^7^, and the gravity and buoyancy of the particles. Among these forces, the interface force is the basis for dust and colloid removal by moving water-air interface^8^,^9^. For the second condition, the small sliding angle is crucial to the rolling behavior of droplets. Thus, self-cleaning can be easily observed on a superhydrophobic surface (SHS). In addition, an autonomous mechanism to achieve self-cleaning on a SHS has been reported, where the contaminants are removed by self-propelled jumping condensate^10^,^11^. However, the durability of the superhydrophobicity of a fabricated surface is questionable now. Once the superhydrophobicity is destroyed by external damage such as scraping, the rolling droplets or the condensate droplets will stop to move and they will adhere to surfaces, resulting in the failure of self-cleaning. Therefore, the self-cleaning ability of superhydrophobic surfaces will be limited.

Droplets impinging is another mechanism for self-cleaning. Droplets impinging is a common natural phenomenon, which has been studied for more than a century. Though large numbers of studies have been concentrated on the morphological evolution of droplets impinging on various surfaces^12^,^13^,^14^,^15^,^16^, detailed investigations on the self-cleaning properties of surfaces by droplet impinging are very few. Furstner^8^ investigated the self-cleaning properties of three superhydrophobic surfaces. He pointed out that water drops with some amount of kinetic impact energy were able to clean these surfaces perfectly. However, only SHSs are investigated and the removal processes of the contaminants are not presented in this work. In reality, superhydrophobic surfaces are less durable, while ordinary surfaces are more common. The SHS would become less hydrophobic with time lapse. If a surface is not superhydrophobic, does the surface still have the self-cleaning ability under the condition of drop impinging? Are there any differences for different types of ducts? To the authors’ knowledge, there have been no published studies devoted to the effects of surface wettability and dust types on the self-cleaning. This area of study is of great importance to the successful realization self-cleaning with less-hydrophobic surfaces.

## Experimental

The method to prepare surfaces with different wettability has been reported^17^. The detailed process is presented in the supporting material. The surface morphology and wettability are characterized by scanning electron microscope (SEM, Fig. S1 in supporting information) and a contact angle system (Dataphysics OCA 20, Fig. S2), respectively. Four surfaces are used, namely, SHS (superhydrophobic surface), HHS (high hydrophobic surface), HS (hydrophobic surface) and GS (glass slide, hydrophilic). They are defined according to their different wettability (advancing and receding contact angles in Fig. S2).

Two types of dust particles are used in this paper. The first type of dust is collected from building site (D1). D1 paticles are common dust particles collected from real environment, which are closely related to our daily life. The investigation of the removal process for these particles has practical significance. D1 are used to approximate the hydrophilic soil fragments. The second is self-prepared epoxy resin microspheres (ERMs)^17^. Their preparation method is shown in supporting information. ERMs are used to approximate the wettability of the hydrophobic plant matter. The properties of the two types of dust particles are analyzed by a variety of analyzing methods, including the SEM (Fig. S3), EDS (Energy-dispersive X-ray spectroscopy, Table S1), FTIR (Fourier transform infrared, Fig. S4) and XRF (X Ray Fluorescence, Table S2).

The self-cleaning experiments are conducted by impinging water droplets on inclined dusty surfaces. The droplet diameter is kept constant at 2.84 mm. Since the surfaces investigated here are oblique, two Weber numbers should be defined. One is a normal impact Weber number (We_N_), the other one is a tangential impact Weber number (We_T_)^15^ as


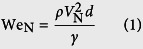



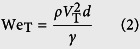


where *ρ, d*, and *γ* are the density, diameter, and surface tension of the drop, respectively. *V*_N_ is a normal impact velocity and *V*_T_ is a tangential impact velocity. Employing the normal impact Weber number is important for investigation of impact dynamics on oblique surfaces^12^,^15^.

## Results and Discussion

### The attachment of dust particles to the water-air interface formed by droplet impinging

**Figure 1:**
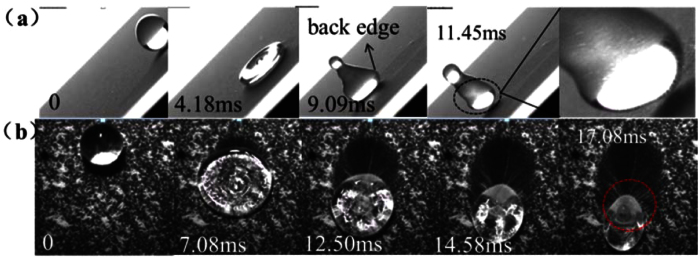
The two types of dust particles picked up by the water-air interfaces at the trailing edge of drops. (**a**) D1 particles on the SHS with We_N_ of 16.8. (**b**) ERMs on the SHS with We_N_ of 23.6. The dusty surfaces are inclined with an angle of 45°.

**Figure 2:**
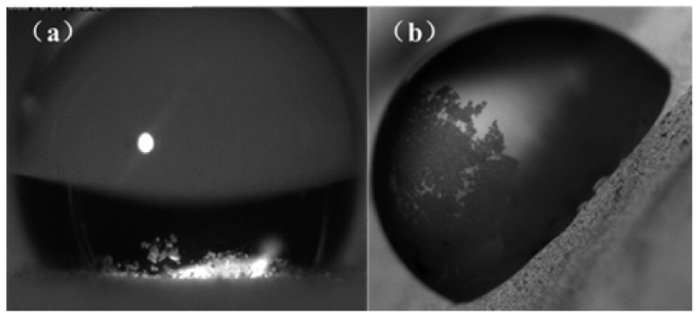
The final states of the two types of particles picked up by the water-air interface if the drops stop moving and retain on the surface. (**a**) D1 particles sink to the bottom of the droplet, (**b**) ERMs adhere to the surface of the droplet, which forms a liquid marble.

The processes of dust particles attaching to the drops are shown in Fig. 1. It can be found that the water-air interfaces formed by the spreading and recoiling behaviors of impinging droplets are the key for the removal process of particles. When a droplet impacts onto an inclined surface, its spreading motion determines the size of the area to be cleared and its recoiling behavior determines the ability to remove particles. It can be found that only the water-air interface at the trailing edge of the droplet can pick up particles efficiently during the recoiling process (the removed particles are indicated by circles in Fig. 1). Then, these particles will move toward the center or to the front of the drop, which is driven by the velocity field inside the droplet. Due to the different property of dust particles, the interactions between the droplets and particles are different. The droplet can totally wet the hydrophilic D1 particles after picked up by the water-air interface at the trailing edge of the droplet (Fig. 1a). These particles then move toward the center of the drop. Finally, these D1 particles would sink to the bottom (Fig. 2a) if the droplet strops moving. The droplet cannot wet the hydrophobic ERMs which only adhere to the surface of the droplet (Fig. 1b). They would move together with the water-air interface. Finally, a liquid marble^18^,^19^ is formed in the local region of the drop after the drop stop moving (Fig. 2b).

### Self-cleaning experiments on superhydrophobic surface (SHS)

**Figure 3:**
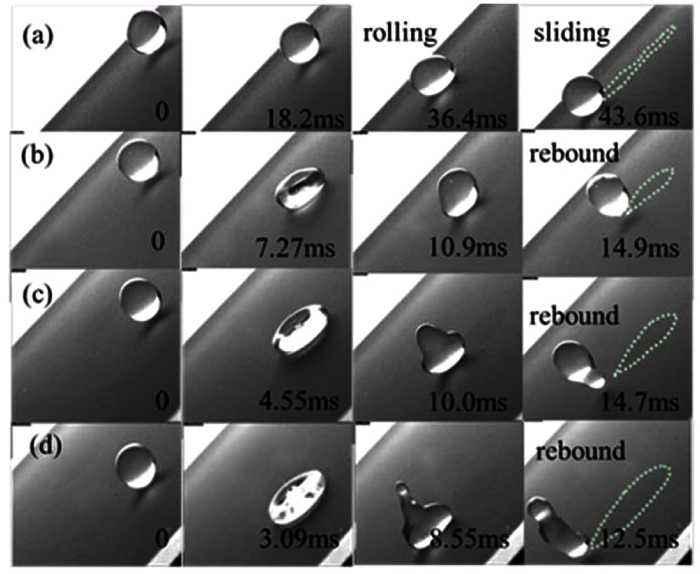
Removal processes of D1 particles on SHSs by droplets impinging under different We_N_: (**a**) We_N_ = 1.14, (**b**) We_N_ = 2.67, (**c**) We_N_ = 8.39, (**d**) We_N_ = 16.8.

Figure 3 shows the different removal processes of D1 particles on the SHS by droplet impinging under different We_N_. The first one is a sliding-rolling removal process when the initial kinetic energy of the droplet is small (Fig. 3a and movie S1). The droplet slides down and takes away the dust particles along the way under the tangential impact force and the gravity. The second is a symmetrical rebound removal process (the shapes at the leading edge and at the trailing edge are similar) under larger We_N_ (We_N_ increases from 2.67 to 16.8 in Fig. 3b–d). The impinging droplets bounce from the surface and pick up particles (movie S2). The higher the We_N_ is, the larger the cleaned region is (marked in Fig. 3 by dash lines). It can be found that the removal processes of ERMs on the SHSs are similar to D1 particles. Under lower We_N_, the sliding-rolling process occurs. If We_N_ increases, ERMs would be removed by the rebound process of drops. Figure S5 shows the same traces for dust removal for the two types of particles. The dynamic processes of drops impinging on clean SHSs are also similar to those on dusty surfaces (Fig. S6). Thus, the wettability of particles have no influences on the self-cleaning of superhydrophobic surfaces. They can be removed either by the sliding-rolling behavior or by the rebound behavior of impinging drops.

### Self-cleaning experiments on high hydrophobic surface (HHS)

**Figure 4:**
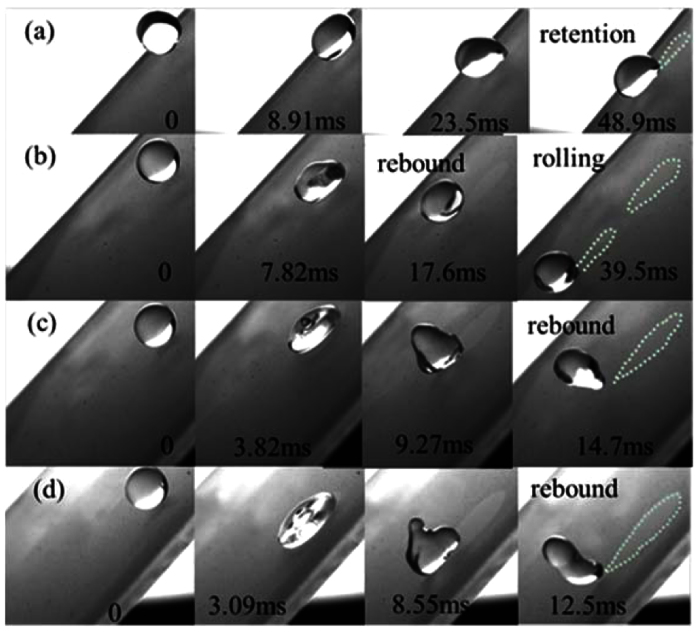
The removal processes of D1 particles on HHSs by droplet impinging under different We_N_: (**a**) We_N_ = 1.14, (**b**) We_N_ = 2.67, (**c**) We_N_ = 8.39, (**d**) We_N_ = 16.8.

Figure 4 shows the removal process of D1 particles on a HHS. A larger initial energy will be needed for the droplet to remove D1 particles. For example, the D1 particles cannot be removed when We_N_ is 1.14 because of the retention of the droplets on the surface. As We_N_ increases to 2.67, the larger initial energy enables the droplet to rebound from the surface and to pick up particles. It can be observed that the rebound height of the droplet is low, resulting in the droplet impacting the surface again with a smaller kinetic energy. Consequently, the D1 particles are then taken away by the rolling motion of the drop. This is called the rebound-rolling removal process. If the We_N_ increases further, the D1 particles are removed by the rebound behavior of droplets (Fig. 4c and d), similar to the cases on the SHS (Fig. 3c and d). The larger initial kinetic energy enables the droplet to rebound far away from the surface. So no rolling motion occurs.

The removal processes of ERMs on the HHS are different from D1 particles. However, they are similar to the ERMs on the SHSs, namely, by the sliding-rolling process at low We_N_ and by the rebound process at high We_N_. The traces of ERMs removal on the HHS under four We_N_ are listed in Fig. S7b. The dusty ERMs surface can be regarded as a superhydrophobic surface^17^, which decreases the surface retention force acting on the droplets. Thus, the removal processes of ERMs on the HHS and SHS are similar. The dynamic processes of drops impinging on clean HHSs are similar to those on dusty HHSs with D1 particles (Fig. S8). This indicates that the ERMs distributed on HHSs really change the dynamic behaviors of drops.

### Self-cleaning experiments on hydrophobic Surface (HS)

**Figure 5:**
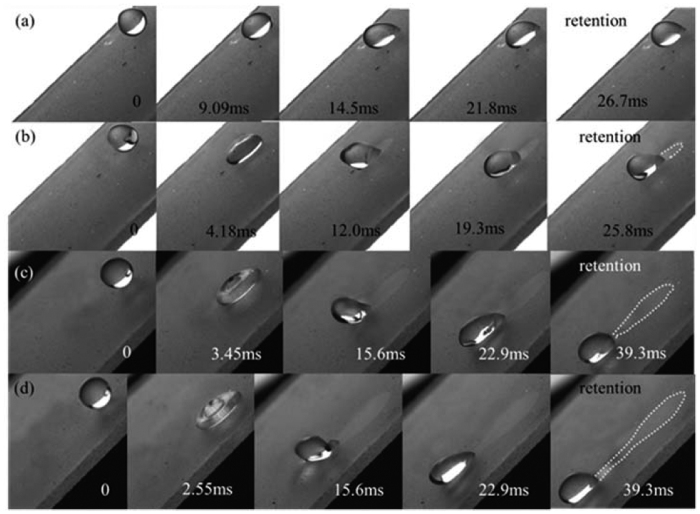
Removal processes of D1 particles on the HS by droplet impinging under different We_N_: (**a**) We_N_ = 1.14, (**b**) We_N_ = 2.67, (**c**) We_N_ = 8.39, (**d**) We_N_ = 16.8.

In Fig. 5, a HS is used. It can be observed that the D1 particles can be picked up by the recoiling behavior of impinging droplets (the cleaned area appears after the droplet moving). The larger the initial kinetic energy of the droplet has (We_N_ ranging from 1.14 to 16.8), the larger the cleaned area appears. However, the droplets eventually retain on the surface after impinging even under high We_N_ (Fig. 5 and movie S3). This is because the small receding angles of HS significantly increase the adherence of droplets. Figure S9 shows the retention states of droplets after picking up D1 particles on the HSs. Thus, the self-cleaning ability of the HS is not well when the dusts are hydrophilic particles.

**Figure 6:**
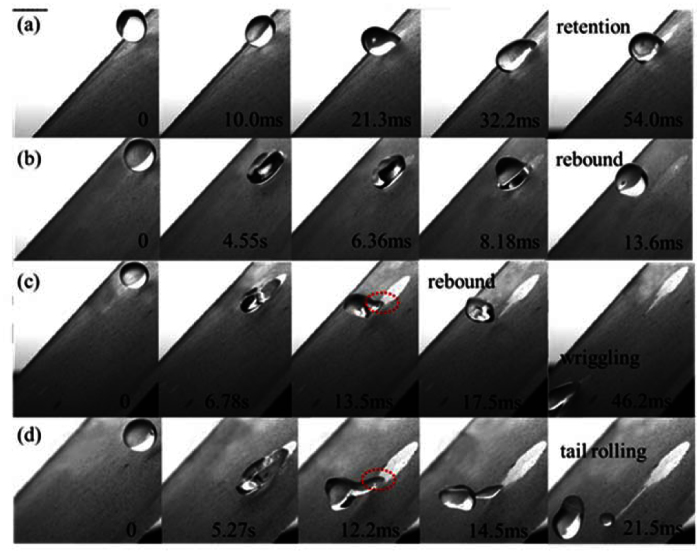
Removal processes of ERMs on HSs by droplet impinging under different We_N_: (**a**) retention, We_N_ = 1.14, (**b**) rebound, We_N_ = 2.67, (**c**) rebound-wriggling, We_N_ = 8.39, (**d**) rebound-tail rolling, We_N_ = 16.8.

For ERMs, their removal processes are quite different from the hydrophilic D1 particles shown in Fig. 6. At low We_N_, the droplet picks up the ERMs and moves downward for a short distance, but eventually it retains on the HS. The small initial energy of the drop cannot overcome the adhesion force of the HS. With an increase in initial kinetic energy, the ERMs can be removal by different processes. At We_N_ = 2.67, ERMs are removed by the rebound behavior of droplet, which is similar to the dust particles on the SHSs (Fig. 3b). If We_N_ increases further, tail sections appear (marked by the cycles in Fig. 6). They pick up ERMs and drag downward along the inclination as the drops attempt to rebound themselves from the surface. Figure 6c is a rebound-wriggling process at We_N_ = 8.39, where the ERMs are initially picked up by the asymmetric rebound behavior (creating a bulge at the leading edge while leaving a thin trail at the back end of the drop), and then by the wriggling motion of the droplet (movie S4). Figure 6d is a rebound-tail rolling process at We_N_ = 16.8, where the ERMs still can be detached by the tail section which breaks up and rolls downward after the main body of the droplet rebounds from the surface (Fig. S9b and movie S5). However, the dynamic processes of drops impinging on clean HSs are similar to those on dusty HSs with D1 particles (Fig. S10). Drops retain on HSs within the scope of the We_N_ of this paper. This also indicates that these hydrophobic ERMs distributed on HSs change the dynamic behaviors of drops.

Through above analysis it can be found that the removal process of ERMs on the HS is also different from the ERMs on the SHS. This is because the formed superhydrophobic layer by ERMs is not permanent. It will be destroyed once the ERMs are picked up by the droplets in the moving process. The naked surface below the particles still influences the removal processes, which will be explained in the following text.

### Self-cleaning experiments on glass slides (GS)

**Figure 7:**
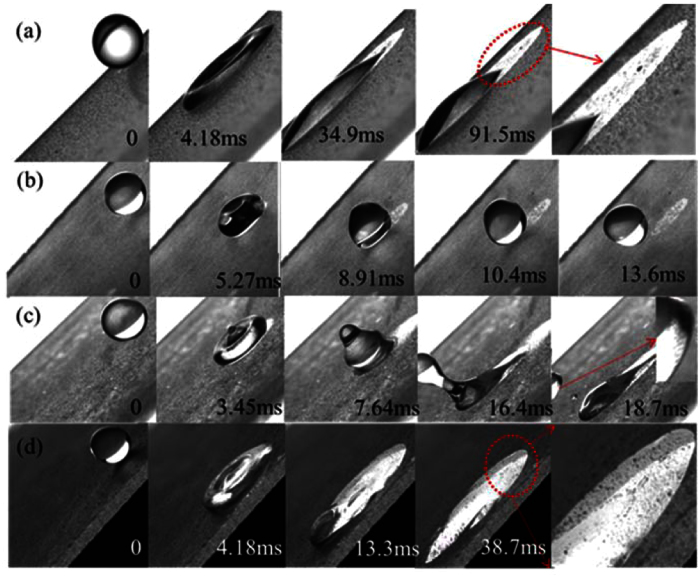
Removal process on a hydrophilic GS surface. (**a**) D1 particles with We_N_ of 16.8. Some particles redeposit on the cleaned area (marked with cycle). (**b–d**) ERMs with different We_N_: (**b**) rebound, We_N_ = 2.67, (**c**) rebound-retention, We_N_ = 8.39, some ERMs can be removed by the drop after it breaks up and rebounds (18.7 ms), (**d**) retention, We_N_ = 16.8.

It can be observed that the particles can attach to the water-air interface when the trailing edge begins to recoil on the GS. However, the cleaned area after the droplet is not so clean due to the redeposition of some attached particles on the surface (Fig. 7a and movie S6). The redeposition of particles may be attributed to the gradual reduction in the receding angle of the trailing edge on the GS. The drop tends to spread and adhere to the hydrophilic surface (Fig. 7a). Therefore, the D1 particles on the GS cannot be removed by droplet impinging (Fig. S11a). Impinging droplets also spread on a clean GS (Fig. S12). Their dynamic behaviors are similar to those on dusty GSs with D1 particles. The rebound behavior of an impinging drop can occur only when the GS is distributed with ERMs, and further, the initial kinetic energy of the drop should be small (Fig. 7b). At lower We_N_, the retention force to the drop is small because the drop contacts with ERMs rather than with the hydrophilic surface. A small fraction of ERMs can be removed by the rebound behavior of drop. As We_N_ increases, the distributed ERMs will be pushed away by the impinging drop, letting the drop to spread on the naked surface. For example, at We_N_ = 8.39, the receding angle of the trailing edge is very large at the early recoiling stage (3.45 ms in Fig. 7c), so a cleaned area appears after the drop. At the later recoiling stage (7.64 ms in Fig. 7c), the receding angle decreases dramatically due to the directly contact of the droplet with the naked hydrophilic GS (movie S7). Eventually, the droplet adheres to the surface. If the We_N_ increases further (16.8, Fig. 7d), the outcome of the impinging drop is similar to the case when the drop impacts on the GS with hydrophilic particles (Fig. 7a). Therefore, the ERMs cannot be removed at high We_N_ (Fig. S11b) on a GS.

**Figure 8:**
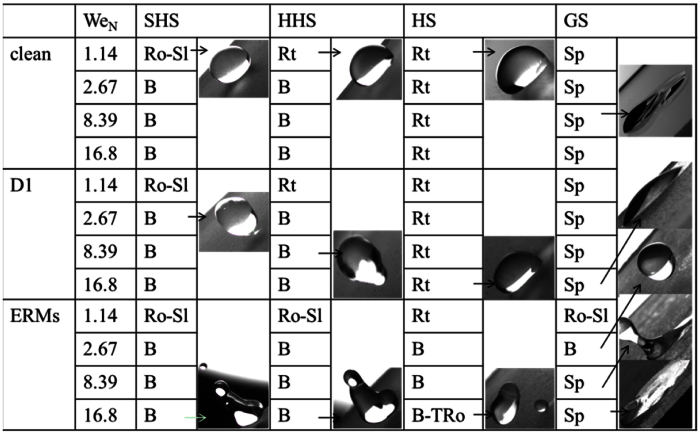
An experimental map of the cleaning action of the droplet under various dynamic and adhesive conditions. B: rebound, B-TRo: rebound-tail rolling, Ro-Sl: rolling-sliding, Rt: retention, Sp: spreading.

The cleaning action of the droplet under various dynamic and adhesive conditions is summarized in Fig. 8. It can be found that the dust types and the wettability of surfaces both influence the self-cleaning. The outcomes of drops impinging on clean surfaces (SHS, HHS, HS and GS) are the same with dusty surfaces distributed with hydrophilic D1 particles. However, the dusty surfaces distributed with ERMs exhibit different self-cleaning actions under the condition of droplet impinging.

## Theoretical considerations

For all these removal mechanisms, the particles are removed by the moving water-air interfaces. In this paper the water-air interface is formed by the spreading and recoiling behavior of a droplet impinging on an inclined surface. The attachment of the particles on the water-air interfaces could be realized, regardless of whether a hydrophilic or hydrophobic sample surface was used. However, the self-cleaning is not realized if the droplets retain on surfaces due to the hysteresis effect. Thus, there are two conditions for self-cleaning: (1) the pickup of particles by the water-air interface, (2) the release of the impinging droplets from the surface. Several forces are involved in the process of particles attaching to the water-air interface.

### Interface force

If a water-air interface is in contact with a solid particle, the net force acting on the particle is the sum of gravity, buoyancy, and interface force. For small particles (radius < 500 *μ*m), the gravity and buoyancy are negligible compared to the interface force^5^,^20^,^21^. The interface force is calculated by^5^,^6^


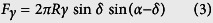


where *R* is the radius of the particle, *γ* is the water/air interfacial tension, *α* is the contact angle of water and particle, and *δ* is the falling angle between water and particle, which determines the position of the water/gas interface on the particle surface^5^. According to the literature^22^, *F*_*γ*_ will reach its maximum when *δ* = (*π* + *α*)/2. Thus, the horizontal component can be obtained as


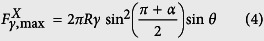


and the vertical component is


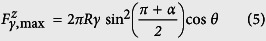


where *θ* is the contact angle of the water-air interface with the solid surface.

### Drag force

Besides the interface force, the water flow can generate a drag force expressed by^5^,^6^


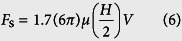


where *μ* is the dynamic viscosity of water, *H* is the water film thickness, and *V* is the velocity of the liquid phase and the water-air interface. However, the drag force will not be a dominant factor compared to the interface force^5^.

### Van der Waals (vdW) force

The adhesion of dust particles on solid surfaces is governed by several forces including van der Waals, electrostatic, gravitational and capillary forces, etc. In general, under the dry and electrically neutral ambient, the van der Waals force can be considered to be the most dominant adhesion force between the particles and solid surfaces^23^,^24^. The experiments in this paper are conducted under the temperature and relative humidity (RH) of 25 ± 2 °C and 45 ± 5%, respectively. In such a dry environment, the capillary force can be ignored (see the detailed explanations in supporting information). In addition, there is no electrostatic potential on samples surfaces (SHSs, HHSs and HSs) measured by an electrostatic measurement instrument (SIMCO-fmx003), the electrostatic force can be ignored. Thus, only van der Waals force is considered in this paper. It can be expressed as^7^,^10^


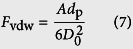


where *A* is the system-dependent Hamaker constant, *d*_p_ is the diameter of a particle, and *D*_0_ is particle-surface separation distance at contact (generally taken to be 0.4 nm)^25^. According to the experimental parameters, the interface force is calculated to be ranging from 10^−7^ to 10^−6^ N, which is larger than the vdW force (<10^−7^) and gravity (<10^−15^). Thus, the particles can be easily picked up by the water-air interface.

Droplets can release themselves from surfaces by the sliding/rolling or the rebound behaviors. The former is inherently a shearing process, where the drop moves on a tilted surface. The latter is tensile process, which is associated with the creation and destruction of interfaces^26^,^27^,^28^.

### Retention force and driven forces when a drop slides/rolls on an inclined surface

The retention force acting on a droplet can be related to the contact angle hysteresis by^28^,^29^,^30^


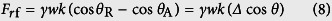



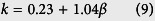



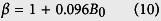



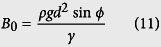


where *w* is the contact width of an impinging droplet. *k* is a numerical constant that depends on the shape of the drop. *θ*_A_ and *θ*_R_ are the advancing and receding angles of water on the inclined surface. *β* is the aspect ratio of contour of the drop. *B*_0_ is the Bond number describing the ratio of gravitational to surface tension forces. *ϕ* is the tilt angle of the sample surface. The contact radius *w* is regarded as the maximum spreading radius of an oblique impacting drop^15^


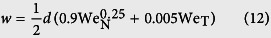


The first term in this equation is the drop maximum spreading due to the first-order normal impact effects^31^, while the second term relates to second-order oblique impact effects. Thus, *F*_rf_ can be expressed as


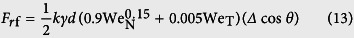


The maximum spreading diameters obtained from Eq. 13 is verified with the experimental data in Fig. S13.

The driving forces (*F*_tol_) that promote the moving of droplet on the inclined surface are gravity force (*F*_g_) and tangential velocity force (*F*_tan_)


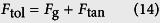


The gravity force is described as


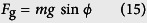


where *m* is the mass of the drop, *g* is the gravitational constant. The gravitational effect also can be described as the form of hydrostatic pressure^15^


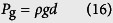


The tangential velocity component of an impinging droplet creates a tangential force *F*_tan_. This force stretching the drop downward upon impact can be associated with the tangential dynamic pressure


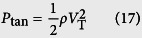


The ratio of the two pressures becomes


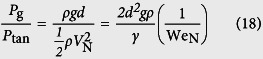


Here, a 45° tilt case is considered (*V*_T_ = *V*_N_). For a droplet diameter of 2.84 mm, Eq. (17) results in an expression of 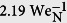. The ratio of gravitational to tangential forces can be obtained as


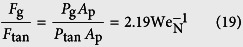


where *A*_p_ is the contact area of the drop on the surface. According to the ratio, the tangential force can be estimated.

### The work of adhesion and the recoiling kinetic energy when a drop rebounds from a surface

For the case of separating the water-solid interface from the solid surface, the work of adhesion could be quantified using^28^


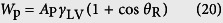


The recoiling kinetic energy (*E*_rk_) of a droplet is obtained from the transition of initial impinging kinetic energy. Due to a lack of comprehensive knowledge about viscous dissipation in the spreading and recoiling stages, the accurate calculation of *E*_rk_ is very hard. A qualitative analysis is presented for the rebound behavior.

### Force analysis

**Figure 9:**
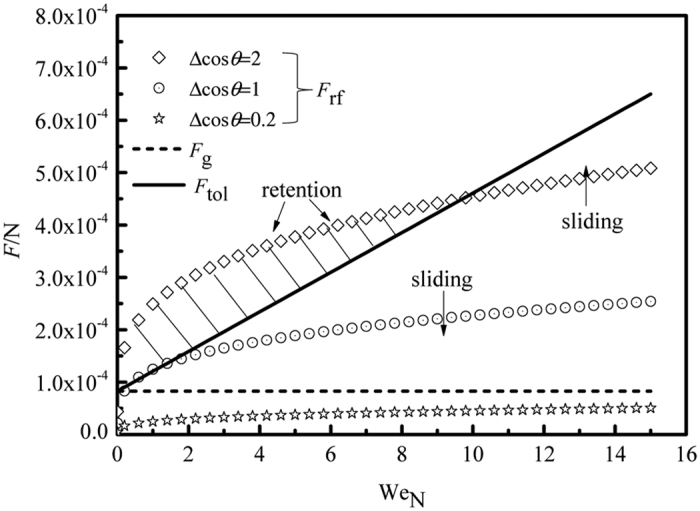
Theoretical values of *F*_rf_, *F*_tol_ and *F*_g_ as a function of We_N_. To simplify the problem, three values of (i.e., 2, 1 and 0.2) are considered to calculate *F*_rf_.

The critical condition for the onset of sliding is given by the balance of the retention force and the driving forces including the gravitational and the tangential forces shown in Fig. 9. The maximum value of *F*_rf_ is obtained at Δcos *θ* = 2, while the minimum value is 0 at Δcos *θ* = 0. However, the latter situation is very ideal and does not exist in reality. To simplify the problem, three values of Δcos*θ* (i.e., 2, 1 and 0.2) are considered to calculate *F*_rf_. It can be observed that the gravity is always larger than the retention force on a surface of small contact angle hysteresis (Δcos *θ* <= 0.2 on superhydrophobic surfaces). A drop can roll on this kind of surface even though no kinetic energy is given. Thus, the two types of particles distributed on SHSs (see experiments in Fig. 3 and Fig. S5) can be easily removed by the sliding-rolling behavior of drops at small We_N_. As shown in Fig. 9, even on a surface with larger hysteresis (Δcos *θ* closes to 2), the *F*_tol_ of a drop can be larger than the *F*_rf_ as long as a certain amount of kinetic energy is given to the drop. The triggering of the drop sliding on surfaces is easy (see the cleared areas in Figs 3, 4, 5, 6 and 7). However, not all these sliding drops can release themselves from surfaces (Figs 5 and 7). This is because the tangential force *F*_tan_ of the drop decreases gradually in the spreading process. Upon impact, the impact kinetic energy of the drop is converted to surface energy and viscous dissipation energy^32^. The tangential velocity of the drop in the spreading stage is reduced. In the recoiling stage, the surface energy of the spreading drop is converted to recoiling kinetic energy and dissipation energy. The tangential velocity of the drop at the trailing edge points upward, which hinders the drop from moving downward. Hence, on a surface with larger retention force (such as the HS in Fig. 5(b–d) or the GS in Fig. 7), the impinging drop would eventually retain on the surface. It can be concluded that the sliding-rolling removal process can be realized at the case of *F*_rf_ < *F*_g_. At the beginning of drop impinging, if *F*_rf_ larger than *F*_g_, but smaller than *F*_tol_ (*F*_rf_ > *F*_g_, *F*_rf_ < *F*_tol_), the drop moves for a short distance. However, it will eventually retain on the surface (due to the gradually decreased *F*_tan_). If *F*_rf_ > *F*_g_ and *F*_rf_ > *F*_tol_, the impinging drop retains on the surface from the beginning. No clean area was left behind it (Fig. 5a).

A drop can release itself from surface by the rebound behavior if the recoiling kinetic energy can overcome the work of adhesion. On the SHS (Fig. 3) or the HHS (Fig. 4), the receding angles of the drops are large, the works of adhesion acting on drops are small. Therefore, the D1 particles on SHS or HHS can be removed by the rebound behavior of droplet under larger We_N_. While on the HS or the GS (shown in Figs 5 and 7a), the receding angles at the trailing edges of impinging drops are very small. Moreover, the contact areas are very large when the droplets move downward on the inclined HS or GS. This indicates that the works of adhesion acting on the drops for the two surfaces are very large. The droplets on HSs could not rebound and they would eventually retain on the surfaces. While on the GS, the drops tend to spread and adhere to the surface. Thus, the D1 particles distributed on the HS or GS cannot be detached efficiently. The ERMs on the HS or on the GS can be removed by droplet impinging, which may be attributed to the hydrophobicity of ERMs, which decreases the retention force or the work of adhesion. The dusty ERMs surface can be regarded as a superhydrophobic surface. The impinging drops can remove dust particles by the sliding or rebound behaviors.

**Figure 10:**
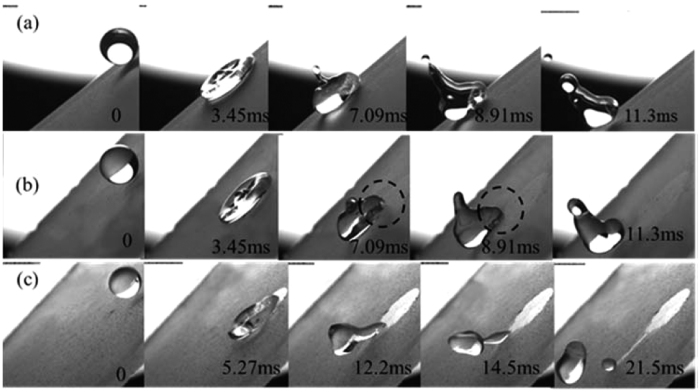
Removal processes of ERMs on the three types of surfaces (**a**) SHS, (**b**) HHS, (**c**) HS.

However, the superhydrophobic layer is not stable and will be destroyed once ERMs are picked up or pushed away by droplets. That is why the ERMs on the GS can only be removed at small We_N_. As mentioned above, only the trailing edge of droplet can pick up particles, resulting in the cleaned area appearing behind the droplet. Thus, the coherent wettability of the sample surfaces mainly influences the trailing edges of droplets. Figure 10 shows the morphologic changes of the droplets picking up ERMs on surfaces with different wettability. On the SHS, the receding angle in the trailing edge is very large, making the droplet rebound from surface quickly. On the HHS, the trailing edge of the moving droplet is pinned (marked with cycles), resulting in a slight decrease in the receding angle. On HS, the naked surface after the droplet will greatly decrease its receding angle at the trailing edge, resulting in a strong adhesive effect acting on the drop. For the leading edge, the effect of surface adhesion is small because of the continuous contact with ERMs. The front section of droplet can be easily separated from the surface in the recoiling stage. As a result of the significant differences in adhesive effect in the leading edge and in the trailing edge, a tail appears in the drop. It finally breaks up in the moving process. The ERMs still can be detached by the tail section which rolls downward after the main body of the droplet rebounds from the surface.

## Conclusions

A detailed study on the self-cleaning of hydrophobic surfaces is presented in which the effects of surface wettability and the types of particles are considered. There self-cleaning is realized by two ways: (1) the pickup of particles by the water-air interface, (2) the release of impinging droplets from the surface. Here, it is disclosed that the self-cleaning can be realized on an ordinary surface by droplet impinging. SHS, HHS, HS and GS are used here to represent different surface wettability. D1 particles are hydrophilic. ERMs are self-prepared hydrophobic particles. Some conclusions can be obtained as follows:

(1) The dust particles can be picked up by the water-air interface at the trailing edges of droplets when the droplets tend to recoil from the inclined surfaces.

(2) The removal processes of the two types of particles on the SHSs are similar, namely, by the sliding-rolling process at low We_N_ and by the rebound process as high We_N_. This indicates that the wettability of particles has no influences on the self-cleaning of superhydrophobic surfaces.

(3) The HHS also has a good self-cleaning ability for the two types of particles. Besides, larger initial kinetic energy will be necessary for droplets to remove hydrophilic dust particles.

(4) The impinging droplets cannot detach hydrophilic dust particles from the HS within the scope of the We_N_ of this paper. However, the hydrophobic ERMs can be removed efficiently from the HS. The tail-like structures appear in the removal processes due to the significant differences in the receding angles between the leading edge and the trailing edge of the drop.

(5) The self-cleaning ability of a GS is poor when hydrophilic dust particles are removed by the impinging droplets. The hydrophobic ERMs can be removed from the GS by the rebound process of drop at small We_N_. The larger kinetic energy is not favorable for the removal process of ERMs on the hydrophilic surfaces.

## Additional Information

**How to cite this article**: Quan, Y.-Y. *et al*. Self-cleaning of Surfaces: the Role of Surface Wettability and Dust Types. *Sci. Rep.* **6**, 38239; doi: 10.1038/srep38239 (2016).

**Publisher's note:** Springer Nature remains neutral with regard to jurisdictional claims in published maps and institutional affiliations.

## Supplementary Material

Supplementary Movie S1

Supplementary Movie S2

Supplementary Movie S3

Supplementary Movie S4

Supplementary Movie S5

Supplementary Movie S6

Supplementary Movie S7

Supplementary Information
