# Supplementary material for: Self-cleaning of Surfaces: the Role of Surface Wettability and Dust Types
Source: Sci Rep. 2016 Dec 5;6:38239. doi: 10.1038/srep38239 (PMC5137015; doi:10.1038/srep38239)
Supplement: Supplementary Information [file srep38239-s8.pdf]

Supporting Information

Self-cleaning of Surfaces: the Role of Surface Wettability and Dust Types

Yun-Yun Quan[1], *Li-Zhi Zhang*[1,2*], *Rong-Hui Qi*[1], *Rong-Rong Cai*[1]

1. Key Laboratory of Enhanced Heat Transfer and Energy Conservation of Education Ministry, School of Chemistry and Chemical Engineering, South China University of Technology, Guangzhou 510640, China
2. State Key Laboratory of Subtropical Building Science, South China University of Technology, Guangzhou 510640, China.

This PDF file includes:
Materials and Methods
Supplementary Text
Figures. S1 to S13
Reference List

Other Supplementary Material for this manuscript includes the following:
Movies S1 to S7

Materials and Methods

Materials

Tetraethoxysilane (TEOS) is purchased from Guangzhou Chemical Reagent Factory. Hexamethyldisilazane (HMDS) is purchased from Aladdin and Adamas. Bisphe-nol Epoxy resin (E-44) and low molecule polyamide curing agent of commercial-grade is purchased from Yueyang petrochemical plant, China. Distilled water is prepared by a Purescience water purification system.

Preparation of Sample Surfaces with Different Wettability

The glass slides are used as hydrophilic surfaces. They are cleaned by ultrasound in a solution mixed with acetone and ethanol to remove the organic contaminants. After that, the slides were thoroughly rinsed with distilled water and dried under vacuum. Hydrophobic and superhydrophobic surfaces are prepared as the previous work [1] .Silica sol (SS) is prepared first. Epoxy resin (0.63 g) and polyamide (0.33 g) are dissolved in silica sol (9 ml) with magnetic stirring until an epoxy suspension (ES) is formed. The ES with different curing time is dripped onto the cleaned glass substrates and are evenly spread out. The resulting glass substrates are then dried at room temperature for one day. After the ethanol evaporates, epoxy resin particular films are formed on the substrates. The wettability of these films changes from hydrophobicity to superhydrophobicity as the curing time of ES prolongs.

Dust Particles

Two types of dust particles are used here. The first type of dust is collected from building site (D1). The second is self-prepared epoxy resin microspheres (ERMs). Their preparation method is as follows [1]. The curing time of ES are prolonged to 11h. The microspheres are collected by centrifuging the ES, then washed with ethanol several times and dried in 60 ℃ for 24h. The ERMs are hydrophobic with diameters in the range of 2-8 μm. The morphology of the two dust particles are shown in the following text. The dusty sample surfaces are prepared by the free settling method. First, the samples are placed horizontally on the bottom of a container. Then, the D1 particles or ERMs are scattered into air by a stirring device. They will settle on the sample surfaces by gravity for a long time. The free settling process is lasted for 24h.

Characterization

The morphology of sample surfaces and dust particles are observed by scanning electron microscope (SEM, S-3700N, HITACHI, Japan). Energy-dispersive X-ray spectroscopy (EDS), Fourier transform infrared (FTIR) spectrometry (BRUKER550, Germany) and X Ray Fluorescence (XRF, PANalytical Axios) are performed to measure the components of dust particles. Thermogravimetry (TG, NETZS, Germany) instrument is utilized to calculate the ignition losses of particles. Surface wettability measurements were performed with a Dataphysics OCA 20 contact angle system at ambient temperature. Advancing and receding contact angles of samples are measured both with volume addition/subtraction and by roll off

methods. The drop volume is 6μL for the former method and 10μL for the latter method[2].

Self-cleaning Experiments by Impinging Droplets

Self-cleaning experiments are performed by contaminating the samples, followed by impinging water droplets on these dusty surfaces, and observing the particle removal processes. The experiments are conducted in a room where the temperature and relative humidity (RH) are measured of 25±2 ℃ and 45±5%, respectively. The impinging velocity of a droplet is controlled by changing its falling height from 0.003m to 0.044m. The inclined angles of sample surfaces are 45°. The droplet diameter is kept constant at 2.84 mm. It's density is 997 g/mL and its surface tension is 0.072 N/m.

A high-speed camera (pco. Dimax HS1, CooKe) is used to observe how the impacting droplets carry away dust particles that are initially adhering to the surfaces. The resolution of the high speed camera is 1000 X 1000 pixels. Its recording rate is 5500 fps (frames per second) and the exposure time is 0.168 ms.

Supplementary Text

Sample Surfaces

The surface morphology of samples with different curing time are observed by SEM (Figure. S1). As the curing time prolonged, the microspheres gradually appear and grow up. The aged silica sol deposited on microsphere surfaces forms the multilevel structures. The wettability of sample surfaces is controlled by adjusting the reaction time of ES. The advancing θ_A, receding θ_R contact angles, and contact angle hysteresis ($\Delta\theta=\theta_A-\theta_R$) are measured[3] when the curing time of ES is changed from 1h to 24h (Figure. S2). As the curing time prolongs, the two angles increase and the contact angle hysteresis decreases, which means that the surfaces become more hydrophobic. For curing time less than 5h, the θ_A of these samples are larger than 130°. However, their receding angles are less than 90°, which means that the hydrophobicity of these samples are poor. They can be regarded as ordinary hydrophobic surfaces. After 9h, the θ_R of samples are larger than 135°, which can be regarded as a threshold for superhydrophobicity. Antonini[3] pointed out that the receding angle greater than 135° used as a threshold for superhydrophobicity will be more rational than the conventionally reported static contact angle greater than 150°. For the convenience of description, we define four kinds of surfaces according to the different wettability (see Figure. S2). The first is the hydrophobic surface (HS, the curing time is less than 5h) with a receding angle smaller than 90°. The second is high hydrophobic surface (HHS, the curing time is more than 5 h and less than 9 h) with a receding angle larger than 90° and smaller than 135°. The third is superhydrophobic surface (SHS, the curing time is larger than 9h) with a receding angle larger than 135°. The last one is the hydrophilic glass slide (GS) without coatings.

Dust Particles

The morphology of the two types of dust particles are observed by SEM. Figure. S3 shows that the

shapes of dusts from building site are irregular, the ERMs are spherical. The dust particles can be distributed evenly on the sample surfaces by free settling method (Figure. S3 c-d).

The components of dust particles are analyzed by EDS, FTIR and XRF. Table S1 shows the types of elements for D1 and ERMs by EDS. ERMs are the curing products of epoxy and polyamide curing agent. They are organic particles which only contain elements of C, O and N (the element of N is not listed in Table S1). These particles are hydrophobic, which has been proved in our previous work [1]. For D1 particles, their main elements are O and Si. "Others" in Table 1 refer to the sum of micro-constituents such as Ti, Zn, Sr, and Cl, etc.. FTIR is utilized to analyze the organic compounds of ERMs and D1 particles in Figure. S4. For ERMs, the absorption peaks at around 3280 cm^{-1}, 2923cm^{-1} and 1607-1495 cm^{-1} are assigned to the acylamino, methylene and benzene groups, respectively [1]. For D1 particles, the absorption peaks are rarely, which shows that their organic components are less. The absorption peaks at around 1043 cm^{-1} cm^{-1} can be considered as SiO_2 [4] .

The D1 particles collected from building sites are mainly inorganic matter analyzed by XRF. The main component for D1 is SiO_2, followed by CaO and Fe_2O_3. The details of the inorganic matters are listed in Table S2. "Others" refer to the sum of micro-constituents such as TiO_2, ZnO and SrO, etc..

Capillary Force

The dust removal experiments are conducted in a room where the temperature and relative humidity (RH) are measured of 25±2 ℃ and 45±5%, respectively.

The liquid bridge is not formed if both the contacting surfaces are hydrophobic[5,6]. Thus, when hydrophobic ERMs particles contact with hydrophobic surfaces (HS, HHS, SHS), the capillary force is not considered.

For hydrophilic particles contacting with hydrophobic surfaces, the total adhesion forces between the two kinds of surfaces are found to be almost constant for all RHs[5,7]. This is because the capillary condensation is weak between surfaces, resulting in small capillary forces. Thus, when hydrophilic D1 particles contact with hydrophobic surfaces (HS, HHS, SHS), the capillary force can be ignored.

If the two contacting surfaces are both hydrophilic (e.g., D1 and glass surface), the effect of RH on the adhesion force is significant. There is no critical RH for capillary condensation, but the adhesion tends to rise steeply at RH > 60%[5,8]. Under the condition of low RH (45%), the capillary force is still small[9,10]. In addition, as shown in Figure 7a and Figure S12, the impinging drops cannot release themselves from glass surfaces. That's to say, D1 particles cannot be carried away by drops no matter whether their adhesion forces are large or small. The adhesion forces of D1 particles have no influence on the results of dust removal process.

Thus, under current conditions, only van der Waals force is considered.

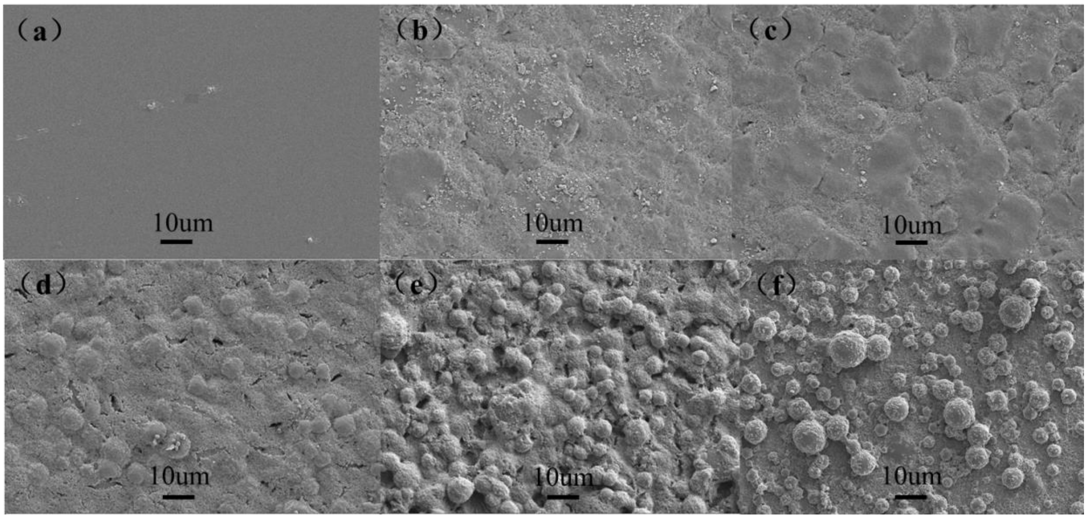

Figure S1 SEM of epoxy resin surfaces with different curing time. (a) 1h, (b) 2h, (c) 3h, (d) 6h, (e) 11h, (f) 24h.

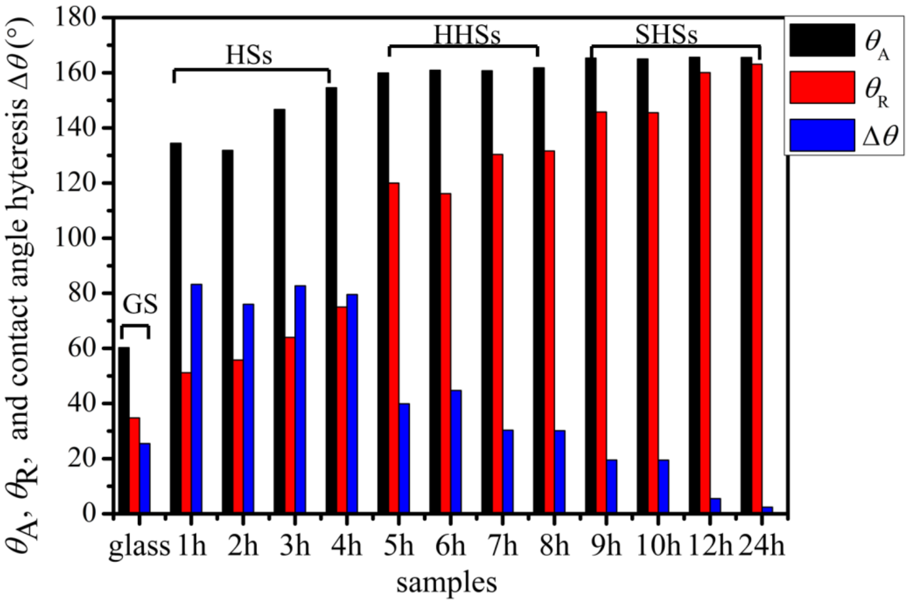

Figure S2 Advancing θ_A, and receding contact angles θ_R, and contact angle hysteresis ($\Delta\theta$) for epoxy resin particular films with different curing time

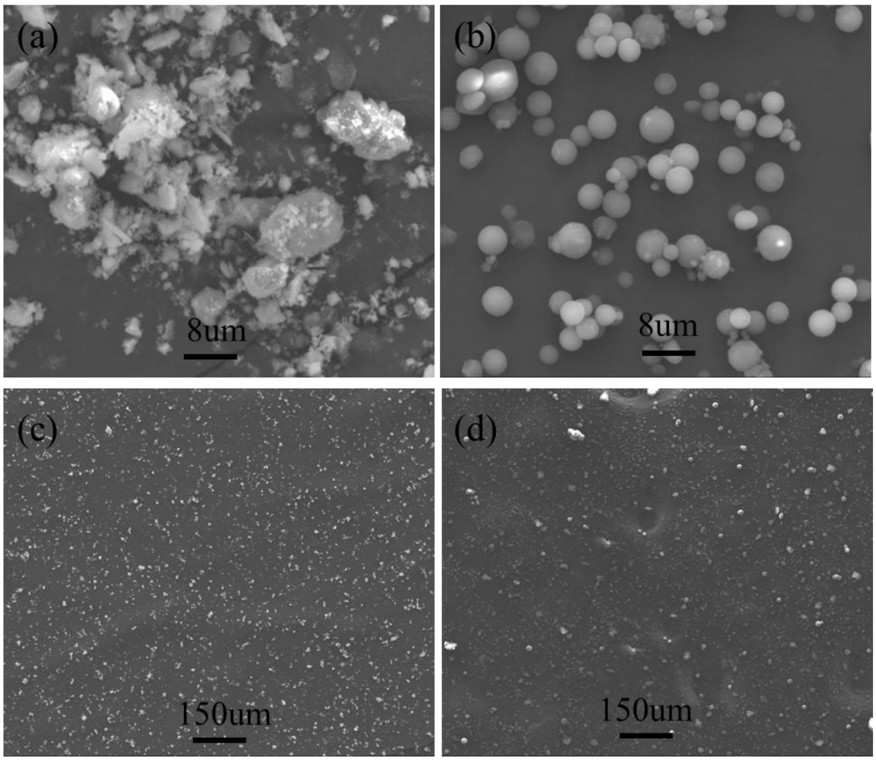

Figure S3 SEM images of the dust particles. (a, c) D1 particles from building site, (b, d) ERMs. The dust particles are spread to the sample surfaces evenly by free settling method (c-d).

Table S1 Elements compositions for the two types of dust analyzed by EDS

dust	C	Si	O	Ca	Na	Mg	K	Fe	P	S	Al	others
D1	11.59*	40.92	45.06	0.74	0.44	0	0.20	0.34	0	0	0.71	0
ERMs	69.96	0	29.46	0	0	0	0	0	0	0	0	0

*Element weight percentage (%) , the sum of all elements for each dust particle is 100

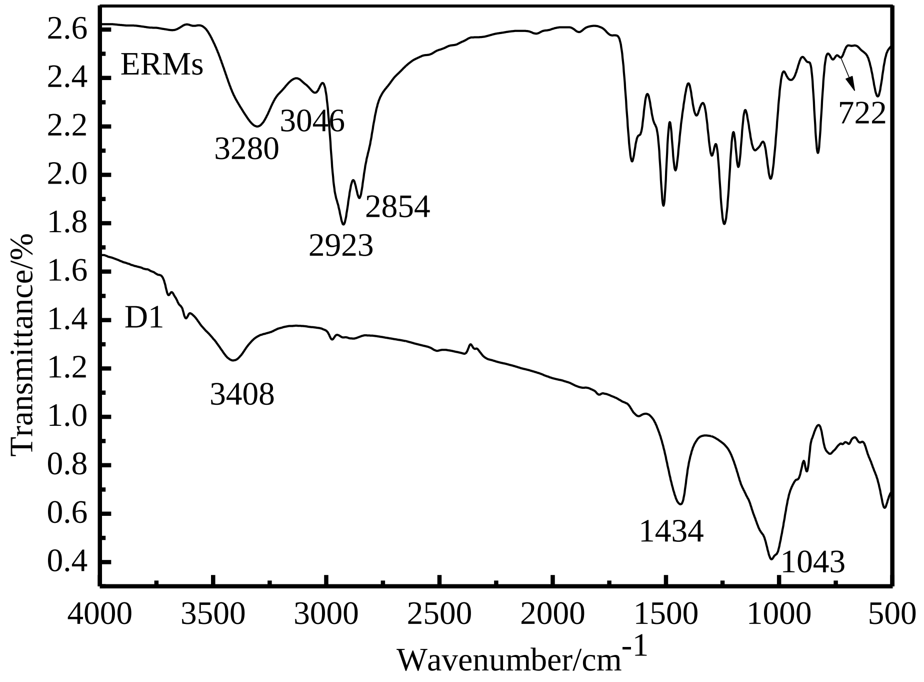

Figure S4 FTIR spectra of D1 and ERMs

Table S2 Inorganic compositions for D1 particles analyzed by XRF

dust	SiO_2	CaO	Fe_2O_3	Na_2O	K_2O	Al_2O_3	SO_3	P_2O_5	MgO	Other
D1	77.63*	3.44	2.00	0.89	4.18	10.68	0.34	0.10	0.33	0.41

*Element weight percentage (%)

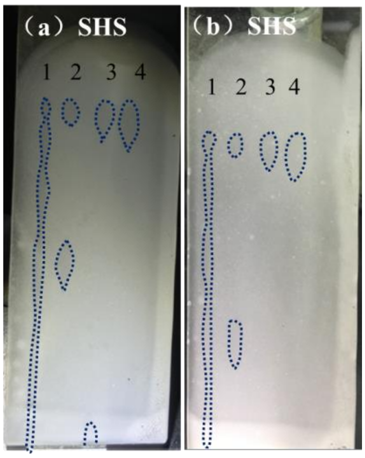

Figure S5 The traces (marked by dotted lines) of the two types of dust particles removed by droplet impinging on SHSs: (a) D1 particles, (b) ERMs. For each surface, points 1, 2, 3 and 4 corresponds to We_N of 1.14, 2.67, 8.39, 16.8, respectively.

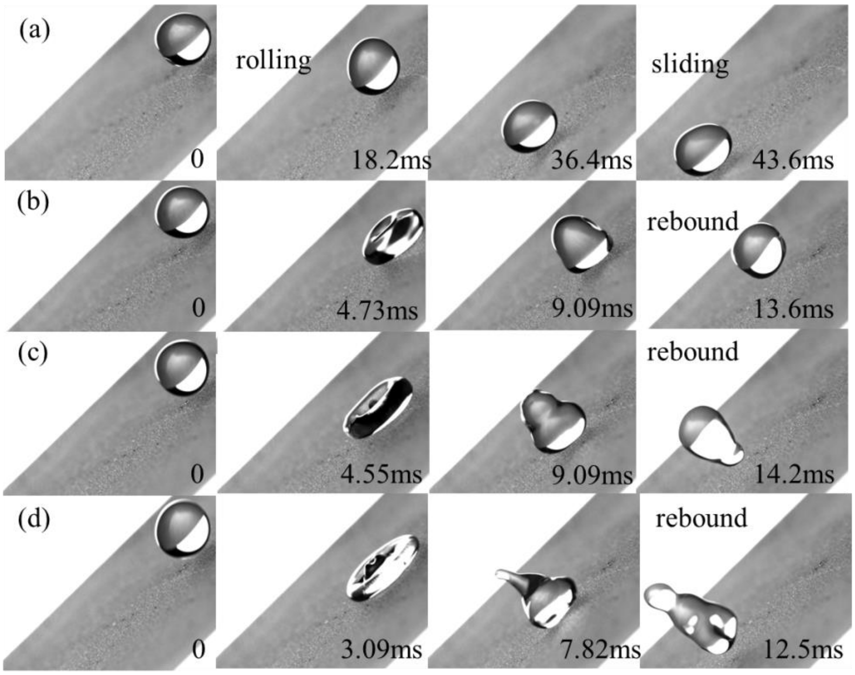

Figure S6 Droplets impinging processes on clean SHSs with different We_N. (a) 1.14, (b) 2.67, (c) 8.39, (d) 16.8.

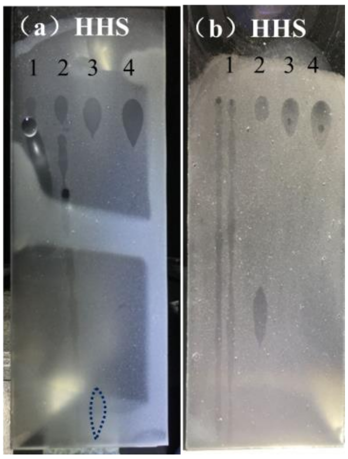

Figure S7 The traces of the two types of dust particles removed by droplet impinging on HHSs: (a) D1 particles, (b) ERMs. For each surface, points 1, 2, 3 and 4 corresponds to We_N of 1.14, 2.67, 8.39, 16.8, respectively.

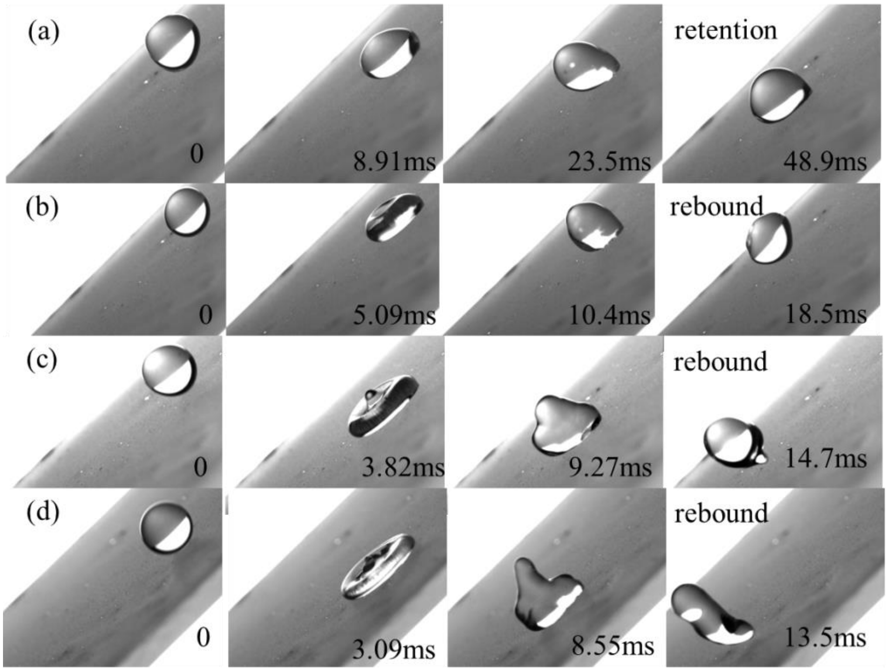

Figure S8 Droplets impinging processes on clean HHSs with different We_N. (a) 1.14, (b) 2.67, (c) 8.39, (d) 16.8.

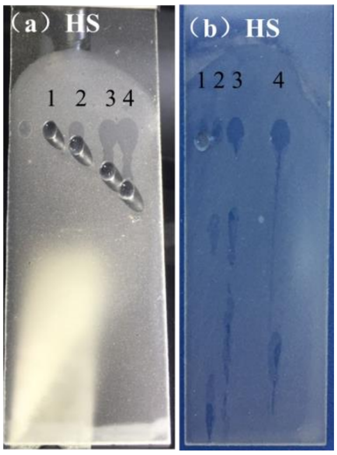

Figure S9 The traces of the two types of dust particles removed by droplet impinging on HSs: (a) D1 particles, (b) ERMs. For each surface, points 1, 2, 3 and 4 corresponds to We_N of 1.14, 2.67, 8.39, 16.8, respectively.

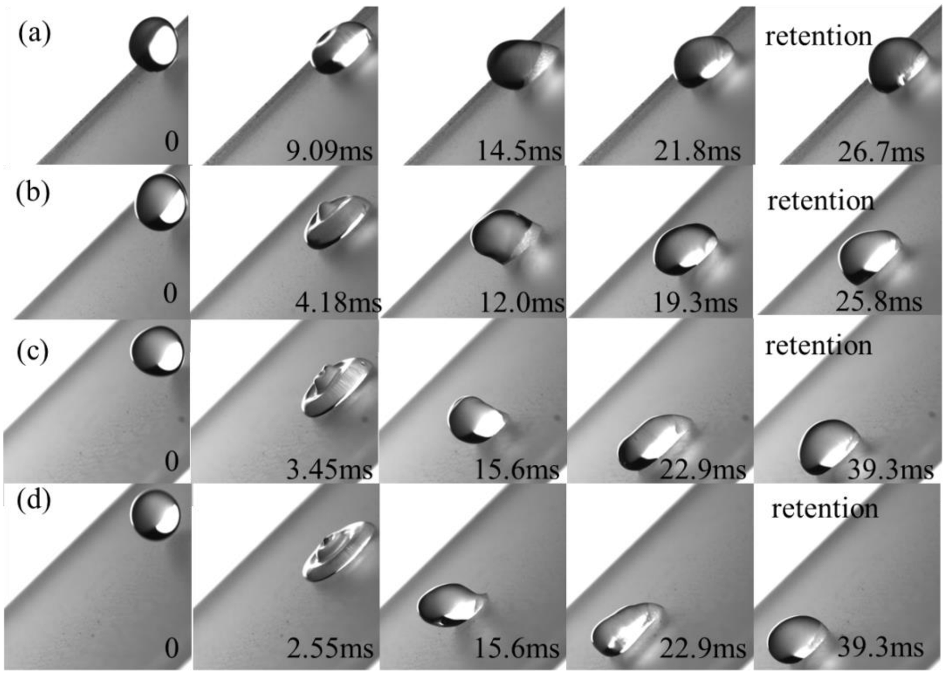

Figure S10 Droplets impinging processes on clean HSs with different We_N. (a) 1.14, (b) 2.67, (c) 8.39, (d) 16.8.

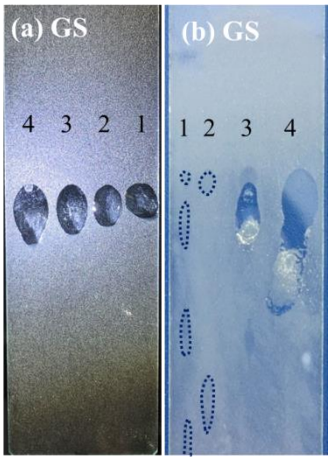

Figure S11 The traces of the two types of dust particles removed by droplet impinging on GSs: (a) D1 particles, (b) ERMs. For each surface, points 1, 2, 3 and 4 corresponds to We_N of 1.14, 2.67, 8.39, 16.8, respectively.

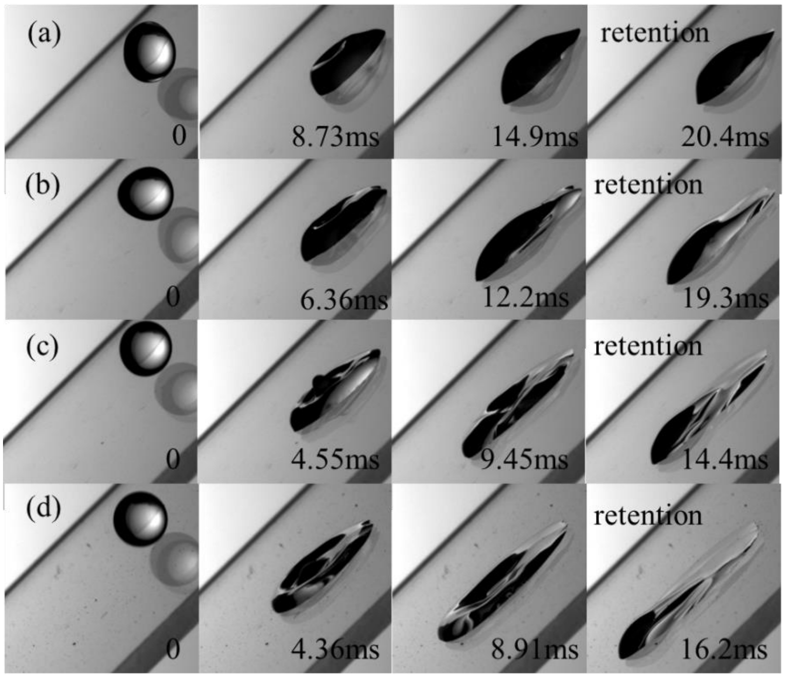

Figure S12 Droplets impinging processes on clean GSs with different We_N. (a) 1.14, (b) 2.67, (c) 8.39, (d) 16.8.

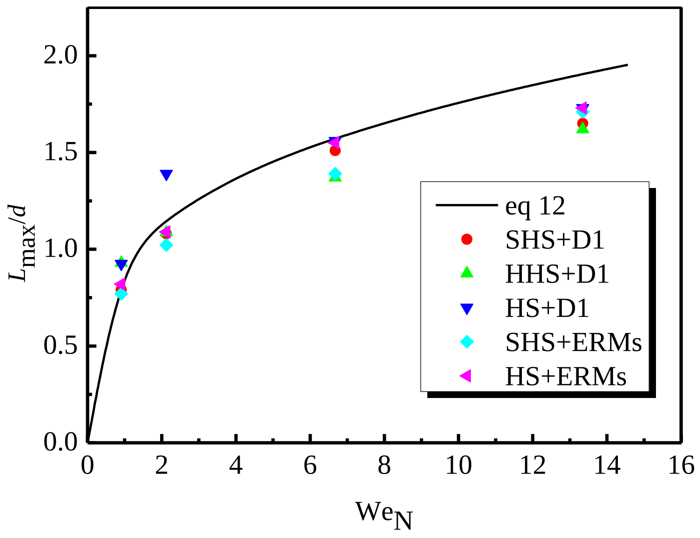

Figure S13 The verification of the maximum spreading diameters obtained from eq 12 with the experimental data

Movie S1
The sliding-rolling removal process occurs on a SHS distributed with D1 particles. The We_N is small (1.14).

Movie S2
The rebound removal process occurs on a SHS distributed with D1 particles. The We_N is 16.8.

Movie S3
The retention of the drop impinging on a HS distributed with D1 particles. The We_N is 8.39.

Movie S4
The rebound-wriggling removal process occurs on a HS distributed with ERMs. The We_N is 8.39.

Movie S5
The rebound-tail rolling removal process occurs on a HS distributed with ERMs. The We_N is 16.8.

Movie S6
The retention of a droplet impinging on a GS (hydrophilic surface) distributed with D1 particles. The We_N is 16.8. Some particles redeposit on the cleaned area, which can be observed clearly form the movie.

Movie S7
The rebound-retention process of a droplet impinging on a GS (hydrophilic surface) distributed with ERMs. The We_N is 8.39. The receding angle on the trailing edge is very large in the early recoiling stage, and a cleaned area appears after the drop. After that, the receding angle decreases dramatically due to the direct contact of the droplet with the hydrophilic GS. A small fraction of ERMs can be removed by the rebound part of the drop.

REFERENCES

1 Quan, Y.-Y. & Zhang, L.-Z. Facile fabrication of superhydrophobic films with fractal structures using epoxy resin microspheres. *Applied Surface Science* **292**, 44-54, doi:10.1016/j.apsusc.2013.11.060 (2014).

2 Scarratt, L. R., Hoatson, B. S., Wood, E. S., Hawkett, B. S. & Neto, C. Durable Superhydrophobic Surfaces via Spontaneous Wrinkling of Teflon AF. *ACS applied materials & interfaces* **8**, 6743-6750 (2016).

3 Antonini, C., Villa, F., Bernagozzi, I., Amirfazli, A. & Marengo, M. Drop rebound after impact: the role of the receding contact angle. *Langmuir* **29**, 16045-16050 (2013).

4 Hollenstein, C. *et al.* Silicon oxide particle formation in RF plasmas investigated by infrared absorption spectroscopy and mass spectrometry. *J. Phys. D: Appl. Phys.* **31**, 74-84 (1998).

5 Fuji, M., Machida, K., Takei, T., Watanabe, T. & Chikazawa, M. Effect of wettability on adhesion force between silica particles evaluated by atomic force microscopy measurement as a function of relative humidity. *Langmuir* **15**, 4584-4589 (1999).

6 Opitz, A., Scherge, M., Ahmed, S. I. U. & Schaefer, J. A. A comparative investigation of thickness

measurements of ultra-thin water films by scanning probe techniques. *Journal of Applied Physics* **101**, 064310, (2007).
7 Fukunishi, A. & Mori, Y. Adhesion force between particles and substrate in a humid atmosphere studied by atomic force microscopy. *Advanced Powder Technol* **17**, 567-580 (2006).
8 Jones, R., Pollock, H. M., Cleaver, J. A. S. & Hodges, C. S. Adhesion forces between glass and silicon surfaces in air studied by AFM: effects of relative humidity, particle size, roughness, and surface treatment. *Langmuir* **18**, 8045-8055 (2002).
9 Tan, C. L. C., Gao, S., Wee, B. S., Asa-Awuku, A. & Thio, B. J. R. Adhesion of dust particles to common indoor surfaces in an air-conditioned environment. *Aerosol Science and Technology* **48**, 541-551, (2014).
10 Thio, B. J. & Meredith, J. C. Measurement of polyamide and polystyrene adhesion with coated-tip atomic force microscopy. *Journal of colloid and interface science* **314**, 52-62 (2007).
